# *N*-Phenylpropyl-*N*′-(3-methoxyphenethyl)piperazine (YZ-185) Attenuates the Conditioned-Rewarding Properties of Cocaine in Mice

**DOI:** 10.1155/2013/546314

**Published:** 2013-09-05

**Authors:** Andrew S. Sage, Scott C. Vannest, Kuo-Hsien Fan, Matthew J. Will, Susan Z. Lever, John R. Lever, Dennis K. Miller

**Affiliations:** ^1^Department of Psychological Sciences, 203 McAlester Hall, University of Missouri, Columbia, MO 65211, USA; ^2^Department of Chemistry, University of Missouri, Columbia, MO 65211, USA; ^3^MU Research Reactor Center, University of Missouri, Columbia, MO 65212, USA; ^4^Research Service, Harry S. Truman Memorial Veterans' Hospital, Columbia, MO 65201, USA; ^5^Departments of Radiology, and Medical Pharmacology and Physiology, University of Missouri, Columbia, MO 65211, USA; ^6^Center for Translational Neuroscience, College of Medicine, University of Missouri, Columbia, MO 65211, USA

## Abstract

Sigma receptor antagonists diminish the effects of cocaine in behavioral assays, including conditioned place preference. Previous locomotor activity experiments in mice determined that the sigma receptor ligand YZ-185 (*N*-phenylpropyl-*N*′-(3-methoxyphenethyl)piperazine) enhanced cocaine-induced hyperactivity at a lower (0.1 **μ**mol/kg) dose and dose-dependently attenuated cocaine-induced hyperactivity at higher (3.16–31.6 **μ**mol/kg) doses. The present study investigated the effect of YZ-185 on cocaine's conditioned-rewarding properties in mice. YZ-185 (0.1, 0.316, 3.16, and 31.6 **μ**mol/kg) did not have intrinsic activity to produce conditioned place preference or aversion. A higher (31.6 **μ**mol/kg) YZ-185 dose, but not lower (0.1–3.16 **μ**mol/kg) YZ-185 doses, prevented the development of place preference to cocaine (66 **μ**mol/kg). YZ-185 did not alter the expression of cocaine place preference. To further characterize YZ-185's behavioral profile, its effects in the elevated zero maze and rotarod procedures were also determined; YZ-185 produced no significant change from baseline in either assay, indicating that the sigma receptors probed by YZ-185 do not regulate anxiety-like or coordinated motor skill behaviors. Overall, these results suggest that YZ-185 is a sigma receptor antagonist at the 31.6 **μ**mol/kg dose and demonstrate that sigma receptors can mediate the development of the conditioned-rewarding properties of cocaine.

## 1. Introduction

 The conditioned place preference procedure in rodents is used to study the conditioned-rewarding properties of drugs of abuse (e.g., cocaine) that can contribute to substance abuse and dependence in humans [1, 2]. During the development of conditioned place preference, cocaine injection is paired with a distinct context, and vehicle is paired with a different context. The expression of place preference is examined in a cocaine-free state when the rodent is given free choice access to both contexts. Conditioned place preference (CPP) is defined as an increase in the amount of time spent on the drug paired side (DPS, e.g., cocaine context), relative to the amount of time spent on this same side during a preconditioning trial (CPP score = DPS_postest_  − DPS_pretest_). The development and expression of cocaine-induced conditioned place preference have most frequently been attributed to the drug's efficacy to regulate dopamine signaling in the brain; however, other neural circuits—such as those associated with memory, motor control, and anxiety—play an important role in this effect [3–5].

 Sigma receptors contribute to cocaine's behavioral effects in rodents, including the development and expression of CPP [6–8]. Overall, sigma receptor antagonists attenuated or blocked cocaine's conditioned-rewarding properties [9–12]. For example, coadministration of BD-1047 with cocaine prevented the development of CPP in mice [10]. In a separate study, after the development of cocaine CPP, BD-1047 blocked the expression of cocaine's conditioned-rewarding properties in rats [9]. The sigma ligand alone did not have conditioned-rewarding or conditioned-aversive properties, as there were no differences in CPP induced between rats that administered only BD-1047 and those that administered saline during the conditioning phase [10]. Recently, the *N*-phenylpropyl-*N*′-substituted piperazine sigma receptor ligand SA-4503 (1-[2-(3,4-dimethoxyphenyl)ethyl]-4-(3-phenylpropyl)piperazine) blocked the development of cocaine CPP in rats [13]. SA-4503 is regarded as a sigma receptor agonist; however, it also blocked cocaine-induced hyperactivity in mice [14], an antagonist effect observed with other ligands in its chemical class [15]. These seemingly contradictory results suggest a mixed agonist/antagonist profile of *N*-substituted piperazine ligands.

 YZ-185 (*N*-phenylpropyl-*N*′-(3-methoxyphenethyl)piperazine), a structural congener to SA4503 in that it only lacks the 4-methoxy group on the phenethyl moiety, binds with high affinity to *σ*1 and *σ*2 sigma receptors (*K*_*i*_ values *≈* 1.4 and 10.2 nM, resp.) and blocks convulsions and death induced by a lethal (~198 *μ*mol/kg) cocaine dose in mice [15]. Recently, our laboratory observed that a high YZ-185 dose (31.6 *μ*mol/kg) attenuated cocaine-induced hyperactivity (66 *μ*mol/kg), while a low YZ-185 dose (0.1 *μ*mol/kg) enhanced cocaine-induced hyperactivity [16]. It is possible that YZ-185 functions as a sigma receptor antagonist at the higher dose and an agonist at the lower dose; demonstrating the characteristic mixed agonist/antagonist profile observed in the related compound SA-4503. The goal of the present study was to continue investigating the behavioral properties of YZ-185. The intrinsic conditioned-rewarding properties of YZ-185 and its effect on the development and expression of cocaine-induced conditioned place preference were determined. To further characterize YZ-185, we also evaluated its effect on anxiety-related behavior with the elevated zero maze procedure [17–19] and coordinated motor skill behavior using the rotarod [20, 21].

## 2. Materials and Methods

### 2.1. Drugs and Chemicals

YZ-185 was synthesized as described previously [15]. Cocaine hydrochloride was purchased from Sigma-Aldrich (St. Louis, MO, USA). All drugs were prepared in saline (0.9% w/v) vehicle. Drug doses refer to the free base weight and are presented in *μ*mol units to allow for comparison to other sigma ligands. 

### 2.2. Animals

Male CD-1 mice (Charles River, 20–22 g at arrival) were housed, 4 or 5 mice per cage with standard rodent chow and water available ad libitum. The colony was maintained under a 12 hr/12 hr light/dark cycle, and all experiments were conducted during the light phase of the cycle. All procedures were approved by the Institutional Animal Care and Use Committee of the University of Missouri.

### 2.3. Effect of Cocaine to Induce Place Conditioning

 The dose-response for cocaine to induce conditioned place preference was determined. Place conditioning experiments were performed in acrylic shuttle boxes (48 × 20 × 20 cm). For conditioning sessions, the boxes were divided into two equal-sized compartments (20 × 20 × 20 cm) by partitions. One side had vertical black/white stripes on the four walls, and the floor was a metal mesh. The other side had horizontal stripes on the walls and a metal rod floor. For pre- and posttest sessions, mice were given free access to both sides by removing the partitions. Sides were separated by a center area (8 × 20 × 20 cm) with black walls and a smooth black acrylic floor. An incandescent lamp was placed approximately 105 cm above the box floor and was the only room lighting. To mask sounds from outside the room, white noise was played continuously. A video camera above the maze was connected to a computer, running Any-Maze software (version 4.81, Stoelting Co., Wood Dale, IL, USA), which measured time spent on each side and distance traveled during both pre- and postconditioned test of preference sessions. Side determination was set at a criterion of 75% of body area existing on that side, calculated in real time by the video tracking software. 

 During the preconditioning test session (Day 1), mice were given free access to either side of the box for 20 minutes. Initial preference—defined as the side in which the mouse spent the most time—was determined. Mice were then assigned a DPS in a counter-balanced fashion such that half of the mice had drug paired with their initially preferred side, while the other half received drug on their nonpreferred side. Conditioning assignments followed a counter-balanced fashion over the following six consecutive days, such that half of the mice were given cocaine (16, 33 or 66 *μ*mol/kg (5, 10, or 20 mg/kg)) or saline (control) injection and confined to their DPS for 20 minutes on Days 2, 4, and 6. The remaining half of mice were given cocaine or saline injection and confined to their DPS for 20 minutes on Days 3, 5, and 7. During non-DPS conditioning days, all mice received saline injection and were confined to their non-DPS for 20 minutes. A post-conditioning test following the same procedure as the pre-conditioning test was performed on the day after the last conditioning session (Day 8).

 The dependent variable was a conditioned place preference score (CPP score) defined as the change in the amount of time (in seconds) a mouse spent on the DPS from pretest to posttest (i.e., CPP score = DPS_postest_  − DPS_pretest_). Data were analyzed via analysis of variance (ANOVA) with cocaine dose as a between-groups factor. Newman-Keuls post hoc tests were followed when appropriate (*P* < 0.05). 

### 2.4. Effect of YZ-185 on Conditioned Place Preference

 The ability of YZ-185 to induce place conditioning was determined via the procedures described. However, on three conditioning days, mice (*n* = 7–12 mice/group) received YZ-185 (0.1, 3.16, or 31.6 *μ*mol/kg (0.042, 1.31, or 13.1 mg/kg)) or saline (0 *μ*mol/kg YZ-185) instead of cocaine. Conditioning scores were analyzed via ANOVA with YZ-185 dose as a between-groups factor.

 The effect of YZ-185 on the development of cocaine-induced place preference also was determined via procedures described. However, on three DPS conditioning days, mice (*n* = 8–12 mice/group) were pretreated with YZ-185 (0.1, 3.16, or 31.6 *μ*mol/kg) or saline (0 *μ*mol/kg YZ-185) 15 minutes prior to cocaine (66 *μ*mol/kg) or saline administration. On the three non-DPS conditioning days, all mice received two saline injections. Conditioning scores were analyzed via ANOVA with YZ-185 dose as a between-group factor.

 The effect of YZ-185 on the expression of cocaine-induced place preference was also determined. Procedures for inducing cocaine (66 *μ*mol/kg) place preference were performed as described. However, on the posttest, mice (*n* = 5–13 mice/group) were pretreated with YZ-185 (0.1, 3.16, or 31.6 *μ*mol/kg) or saline (0 *μ*mol/kg YZ-185) 15 minutes prior to being given free access during the post-conditioning test. Conditioning scores were analyzed via ANOVA with YZ-185 dose as a between-group factor.

### 2.5. Effect of YZ-185 on Anxiety Behavior

 To examine effects of YZ-185 on anxiety-related behaviors, additional groups of mice (*n* = 10-11 mice/group) were randomly assigned to receive YZ-185 (0.1, 3.16, and 31.6 *μ*mol/kg) or saline (0 *μ*mol/kg YZ-185) injection. 30 minutes following treatment, mice were placed on an elevated zero maze (Bond Life Sciences Center, Columbia, MO, USA) for 5 minutes. The maze was constructed of grey acrylic in a circular track 10 cm wide, 105 cm in diameter and elevated 72 cm from the floor. The maze was divided into four equal-length arms. Two arms were open, and two arms were bounded by grey acrylic walls 28 cm in height. An incandescent lamp was placed approximately 2 m above the maze and was the only room lighting. White noise was played continuously. A video camera above the maze recorded the location of the mice, and scoring was later completed by an observer blind to the treatment condition. Time in the open arm and the number of entries into the open arm were determined. These two variables were analyzed via separate ANOVA with YZ-185 dose as a between-groups factor.

### 2.6. Effect of YZ-185 on Coordinated Motor Skill Behavior

To examine possible effects of YZ-185 on coordinated motor skill behavior, mice were placed on a rotarod apparatus (ENV-577M, Med Associates, Georgia, VT, USA) consisting of a horizontal rod (3.2 cm diameter) 16.5 cm above the floor of the apparatus. On three consecutive days (Days 1–3), mice (*n* = 10-11 mice/group) were placed on the rod, and its rotation increased at a consistent rate (4–40 cm/min) across a 5-minute session. The duration of time each mouse remained on the rod was recorded. On the following day (Day 4), mice were randomly assigned to receive YZ-185 (0.1, 3.16, and 31.6 *μ*mol/kg) or saline (0 *μ*mol/kg YZ-185) injection and after 30 minutes were placed on the rod. Data from Days 1–3 were analyzed via ANOVA with Day as a within-subjects factor. The data from Day 4 were analyzed via ANOVA with YZ-185 dose as a between-groups factor.

## 3. Results and Discussion

### 3.1. Effect of Cocaine to Induce Place Conditioning

 In the experiment to determine cocaine's efficacy to produce conditioned place preference, analysis of conditioning scores revealed a significant main effect of cocaine dose (*F*(3,68) = 11.80; *P* < 0.001). Post hoc tests revealed that CPP scores were greater for mice that received 16 *μ*mol/kg (mean = 135.4 s; SEM = ±48.2 s), 33 *μ*mol/kg (mean = 123.4 s; SEM = ±52.2 s), or 66 *μ*mol/kg (mean = 148.5 s; SEM = ±20.1 s) cocaine than for mice that received only saline (mean = 49.1 s; SEM = ±24.7 s). There were no significant differences between the three groups of mice that administered cocaine. Thus, cocaine produced conditioned place preference, although the effect was not dose dependent.

### 3.2. Effect of YZ-185 on Conditioned Place Preference

**Figure 1:**
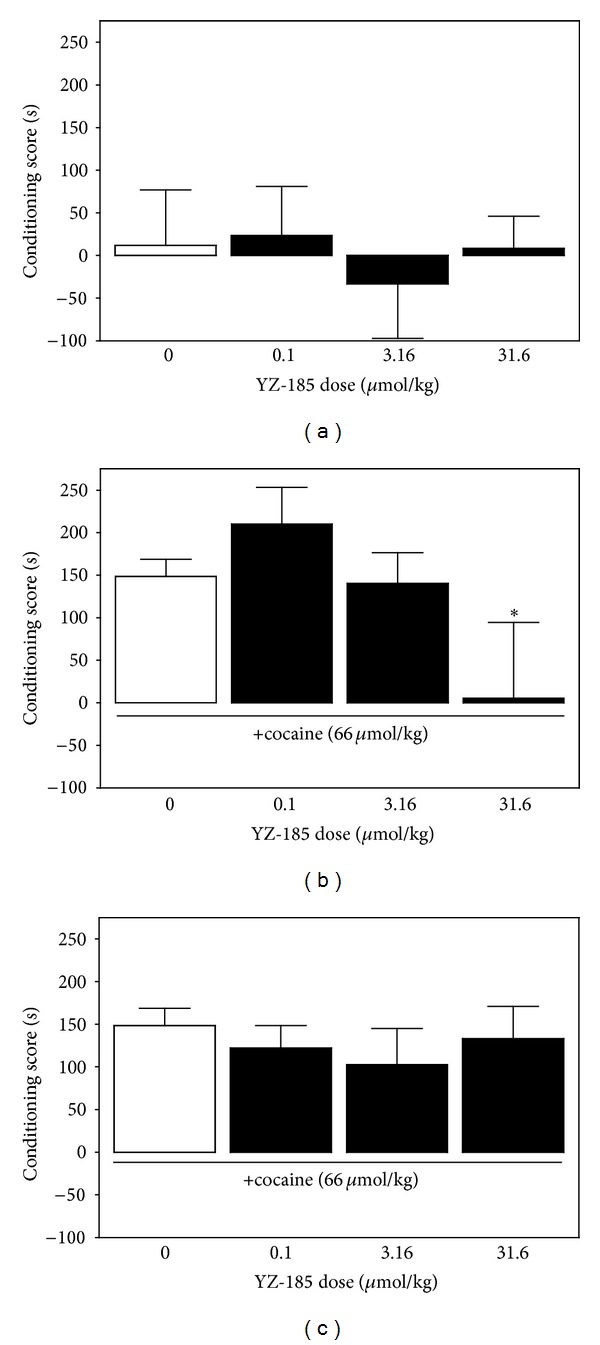
YZ-185 blocks the development of place preference to cocaine, but it does not produce CPP, place aversion, or alter the expression of cocaine's conditioned-rewarding effects. Data represent mean (±SEM) conditioning scores. Panel (a) depicts the lack of intrinsic efficacy of YZ-185 to produce CPP. Panel (b) depicts the effect of YZ-185 on the development of cocaine CPP in mice that received YZ-185 15 minutes prior to cocaine during conditioning sessions. The asterisk indicates a significant difference from the group that received saline (0 *μ*mol/kg YZ-185). Panel (c) represents the effect of YZ-185 on the expression of cocaine place preference in mice that received YZ-185 only on after test.

 The efficacy of YZ-185 to produce a conditioned place preference or aversion was determined, and the data are presented in Figure 1(a). There were no significant differences (*F*(3,34) = 0.77; *P* = 0.52) between the groups of mice that administered YZ-185 (0.1–31.6 *μ*mol/kg) or saline (0 *μ*mol/kg YZ-185), indicating that YZ-185 did not have conditioned-rewarding or conditioned-aversive properties.

Figure 1(b) depicts CPP scores for mice that received concurrent YZ-185 and cocaine administration during conditioning to investigate the development of cocaine CPP. A significant main effect of YZ-185 dose was revealed (*F*(3,36) = 6.32; *P* < 0.01). Post hoc analyses revealed that CPP scores for mice that received 31.6 *μ*mol/kg YZ-185 and cocaine were less than scores for mice that received saline and cocaine. There were no significant differences in CPP scores between mice that received 0.1 *μ*mol/kg or 3.16 *μ*mol/kg YZ-185 and cocaine and mice that received saline and cocaine. Thus, only the high YZ-185 dose blocked the development of CPP to cocaine.

Figure 1(c) depicts conditioning scores for mice that received YZ-185 only on the posttest to investigate the expression of cocaine CPP. There were no significant differences (*F*(3,36) = 0.15; *P* = 0.93) between groups, indicating that the sigma receptor ligand did not affect the expression of cocaine CPP.

### 3.3. Effect of YZ-185 on Anxiety-Like Behavior and on Coordinated Motor Skill Behavior

**Figure 2:**
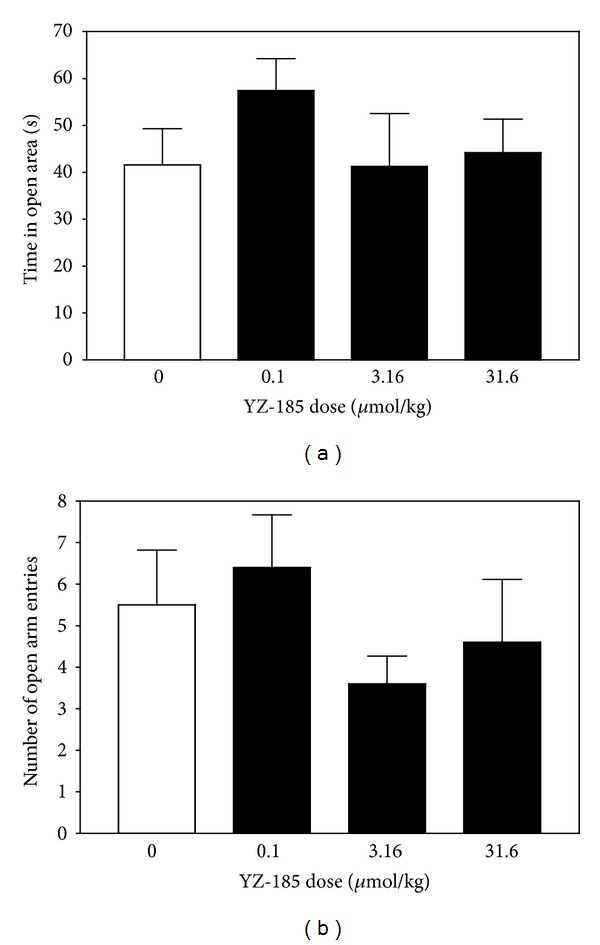
YZ-185 does not alter anxiety-related behavior. Panel (a) presents mean (±SEM) time spent in the open area of the maze, and panel (b) depicts mean (±SEM) total number of entries to the open area.

 The effect of YZ-185 on time spent in the open quadrants of the zero maze and the number of quadrant entries were analyzed and are presented in Figures 2(a) and 2(b), respectively. The main effect of YZ-185 dose was not significant for either dependent variable (*F*(3,42) = 0.71; *P* = 0.56, and *F*(3,42) = 1.15; *P* = 0.34, resp.). Thus, YZ-185 did not affect anxiety-like behavior as assessed by the zero maze.

**Figure 3:**
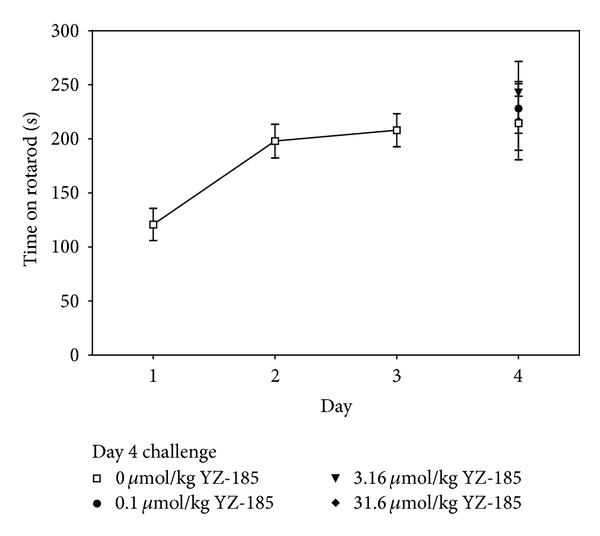
YZ-185 does not alter coordinated motor behavior. Data represent the mean (±SEM) time on the rotarod during (Days 1–3) and after pretreatment with YZ-185 (Day 4).

Figure 3 presents performance on the rotarod. There was a significant increase in time on the rod on Days 2 and 3, relative to Day 1 (*F*(2,82) = 19.24; *P* < 0.001), indicating the development of motor skill learning. However, the main effect of YZ-185 dose from Day 4, when mice received YZ-185 treatment before rod placement, was not significant (*F*(3,42) = 0.50; *P* = 0.68). These data indicate that YZ-185 did not alter the expression of previously learned motor skill behavior.

## 4. Conclusions 

In our previous study [16], 66 *μ*mol/kg cocaine produced hyperactivity that was altered by the *N*-phenylpropyl-*N*′-substituted piperazine sigma ligands tested, including YZ-185. In fact, cocaine-induced hyperactivity was suppressed by 31.6 *μ*mol/kg YZ-185 [16]. Presently, this same cocaine dose induced significant CPP that was sensitive to YZ-185 administration. Pretreatment with 31.6 *μ*mol/kg YZ-185 during conditioning blocked cocaine-induced CPP. In previous CPP studies, other sigma receptor antagonists (e.g., BD-1047 and CM-156) also diminished the development of cocaine's conditioned-rewarding properties [10, 12]. Furthermore, Matsumoto and colleagues [15] reported that this YZ-185 dose range markedly decreased cocaine-induced seizures in mice. This pattern suggests YZ-185 functions as a sigma receptor antagonist, at least at higher doses. Relatedly, Mori and colleagues [13] recently reported that the putative sigma receptor agonist SA-4503, an *N*-substituted piperazine similar to YZ-185, prevented the development of CPP to cocaine in rats. Rodvelt and colleagues [14] also reported that SA-4503 attenuated cocaine-induced hyperactivity. Though initially characterized as a sigma receptor agonist, SA-4503 could be a mixed agonist/antagonist.

Like SA-4503, YZ-185 has also been shown to display agonist properties. The 0.1 *μ*mol/kg YZ-185 dose enhanced cocaine-induced hyperactivity [16]. Presently, 0.1 *μ*mol/kg YZ-185 did not significantly affect the development of CPP to cocaine, but showed a trend toward enhancement. YZ-185 too may have a mixed agonist/antagonist profile that is dose dependent and selective for specific aspects of rodent behavior. Neither 0.1 nor 31.6 *μ*mol/kg YZ-185 induced CPP or altered behavior in the zero maze or rotarod, further indicating a selective behavioral profile.

The development of CPP reflects the association of the unconditioned stimulus (US, cocaine) with the conditioned stimulus (CS, the context) [2, 22]. Sigma receptors may contribute specifically to the interoceptive properties of the US and/or the CS [23, 24] or more globally interfere with the CS-US association. Previous studies where YZ-185 diminished cocaine's effects on locomotor activity [16] and convulsions [15] suggest that YZ-185 diminished cocaine-induced CPP by attenuating the US properties of cocaine (i.e., it diminishes cocaine's efficacy). However, future studies on YZ-185 and other sigma receptor ligands on classical conditioning are warranted [25], considering the prophylactic potential of sigma ligands in models of cognitive diseases [26].

In the absence of cocaine and across the dose range examined, YZ-185 did not induce CPP, indicating that YZ-185 does not have conditioned-rewarding properties like cocaine or other abused drugs [1]. Additionally, the finding indicates that YZ-185 does not have conditioned aversive properties, indicated by reduced time spent on DPS, and thus did not block the development of CPP to cocaine by inducing an aversive state (e.g., malaise or anxiety).

While YZ-185 blocked the development of cocaine CPP, it was ineffective at blocking the expression of the CPP after cocaine's conditioned-rewarding properties were established. Systematic evaluation of sigma receptor ligands on the development versus expression of cocaine place preference has not been reported, although BD-1047 blocked the development in mice [10] and the expression in rats [9]. It is possible that YZ-185 could diminish the expression of CPP established given fewer CS-US pairings (e.g., a single conditioning trial rather than three trials) or given a lower cocaine dose. The regimen used to establish cocaine-induced CPP in the present study was selected based on the cocaine CPP literature [27], and the 66 mol/kg cocaine dose was susceptible to *N*-substituted piperazine treatment in another behavioral measure (i.e., cocaine-induced hyperactivity [16]).

YZ-185 was examined in the zero maze and rotarod assays to further characterize its pharmacological profile and understand potential behavioral mechanisms to alter cocaine's effects. The elevated zero maze is commonly used to measure a drug's anxiolytic/anxiogenic properties [17, 24]. Some sigma receptor ligands diminish anxiety in rodents [28, 29], and these receptors are a clinical target for the development of anxiolytics [26]. YZ-185 did not induce or minimize anxiety-like behaviors in mice, relative to mice treated with saline vehicle suggesting that this ligand lacks efficacy to regulate stress and anxiety. The rotarod test is used to evaluate motor skill learning/memory in rodents and is sensitive to treatment with neurotoxins, including those targeting dopamine pathways in brain [20, 21]. YZ-185 did not alter performance on the rotarod, relative to mice treated with vehicle, consistent with previous work with BD-1047 [30]. This suggests that YZ-185's effects on cocaine-induced hyperactivity and CPP are not related to impairments in motor control.

 The *N*-phenylpropyl-*N*′-substituted piperazine sigma receptor ligand YZ-185 binds with high affinity (*K*_*i*_ values *≈* 1–10 nM) to sigma receptors in brain [15] and blocks the development of cocaine-conditioned place preference at a high (31.6 *μ*mol/kg) dose. This dose also attenuates cocaine-induced hyperactivity in mice [16]. These results suggest that YZ-185 is a sigma receptor antagonist, at least at this dose. The present results also extend previous research suggesting a role for sigma receptors in the development of cocaine's conditioned-rewarding properties [9–12] that can contribute to substance abuse and dependence in humans [1].
